# Monitoring Patellar Tendon Stiffness in Relation to Load, Function and Pain

**DOI:** 10.1055/a-2930-7880

**Published:** 2026-08-31

**Authors:** Lotte van Dam, Rieneke Terink, Inge van den Akker-Scheek, Johannes Zwerver

**Affiliations:** 1 Department of Human Movement Sciences 3647 University of GroningenGroningenNetherlands; 2 Sports Medicine 3096 Gelderse Vallei HospitalEdeGENetherlands; 3 Orthopedics 84792 University of Groningen Faculty of Medical SciencesGroningenGRNetherlands

**Keywords:** tendotonometry, compressive transverse stiffness, early detection, patellar tendinopathy

## Abstract

Monitoring patellar tendon stiffness may provide new insights into tendon adaptation due to loads and pathology. This 12-week prospective observational pilot study aimed to determine the feasibility of monitoring patellar tendon stiffness in relation to training load, pain, and function in male talent volleyball players. Feasibility outcomes included willingness to participate and retention rate, measurement completeness, and adverse events. Willingness to participate and retention rate were 100% in the included 15 volleyball players. Measurement completeness was below the pre-defined success criteria: 72% for stiffness before training and 66% after training; 52% for internal load and 38% for external load; 71% for experienced pain measurements; and 68% for questionnaire completion. This feasibility study indicates that there are several challenges in obtaining a complete data set when monitoring talented volleyball players. Communication between researcher–coach–athlete, feedback on collected data, and education on the importance of maintaining (tendon) health can be considered important factors to overcome these challenges.

## Introduction

Patellar tendinopathy is a common injury among athletes exposed to high jump loads, and stiff landing patterns with an increased tendon load appear to be associated with it. 1​ The injury often causes persistent or recurrent symptoms, adversely affecting sports performance. 2​ While prevalence rates may reach 45% in elite volleyball players, 3​ current treatments do not guarantee full recovery, 4​ with only approximately 45% of patients reporting recovery after 6 months of treatment. 5​ These findings highlight the importance of prevention and early detection strategies.

Preventive strategies emphasize load management. The training load can be adjusted based on individual internal (perceived exertion) and external load metrics (e.g., jump frequency). 6​ However, the load management can be challenging and does not always prevent tendinopathy. To improve load management in the prevention of tendinopathy, monitoring tendon properties, such as tendon stiffness, seems promising. 7​ 8​ 9​ ​10​ Tendon stiffness is known to change due to training and pathology. 11​ Monitoring tendon stiffness could provide information about the (mal)adaptation of tendons to repetitive load.

Tendotonometry is a non-invasive, easy-to-use, and reliable method to measure compressive transverse patellar tendon (PT) stiffness. It calculates stiffness via a digital device that applies a brief impulse to the skin and analyses the resulting oscillations in the underlying tissue. 12​ It can detect changes after short- and long-term training and could potentially detect changes due to pathology. 13​ ​14​ Therefore, it could be a valuable tool in the early detection of symptomatic patellar tendinopathy. However, the practical implementation in an elite athlete setting, where logistical constraints, training schedules, and measurement consistency interfere with monitoring, warrants the evaluation of feasibility. This is needed before embarking on large-scale trials and implementation in sports practice.

Monitoring PT stiffness together with load and pain may provide new insights into the progression of stiffness over time in relation to the development of pathology and pain. To our knowledge, no studies have monitored this before in combination with the practical implementation in an elite athlete setting. Therefore, this prospective observational feasibility and pilot study aimed to determine the feasibility of monitoring PT stiffness over a 12-week period in relation to training load, pain, and function in talented volleyball players.

## Materials and methods

### Study setting

This prospective cohort study in talent Dutch volleyball players followed current pilot and feasibility guidelines. 15​ ​16​ Feasibility outcomes included willingness to participate, retention rate, measurement completeness and adverse events.

**Fig. 1:**
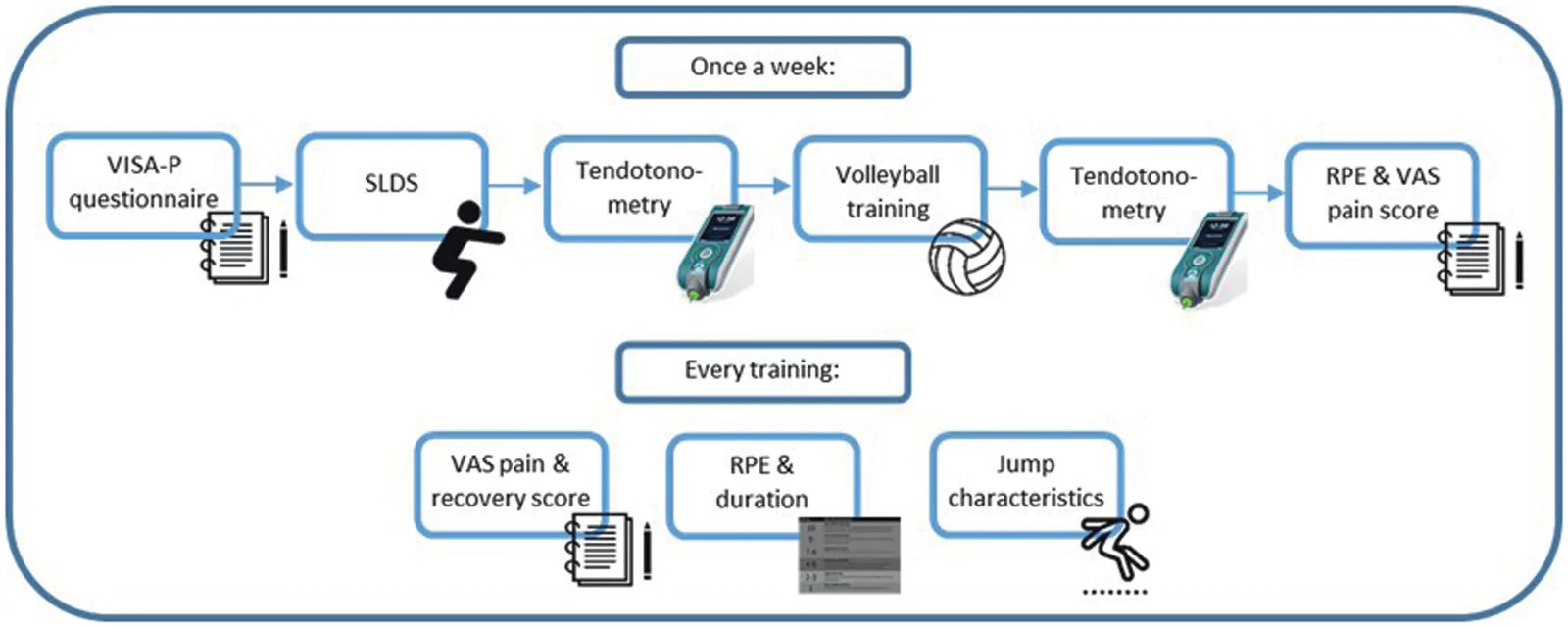
A study overview containing the weekly measurements of PT stiffness measured with tendotonometry and pain assessment tests, as well as measurements of load and pain that took place during every training session. Figure adapted from the study by van Dam et al. 33​ RPE, rating of perceived exertion, SLDS, single-leg decline squat, VAS, visual analogue scale, VISA-P, Victorian Institute of Sports Assessment—Patella.

PT stiffness, training load, pain and function were monitored over a 12-week training period without matches ( Fig. 1 ). Players followed their usual schedule of five ~3-hour sessions/wk. The training load was monitored using questionnaires and measuring jump characteristics. Measurements were performed weekly at the Dutch Olympic Centre between March 1 ^st^ and May 23 ^rd^ , 2023. PT stiffness and patellar tendinopathy symptoms were assessed once a week before and immediately after the Tuesday afternoon training session, and took on average 5 minutes per participant. All measurements took place in the medical examination room next to the training location.

### Ethics

This study adhered to the Declaration of Helsinki (64th WMA Assembly, October 2024). Only observational coded data were used. The Medical Ethics Committee Oost-NL granted a non-WMO declaration on January 27 ^th^ , 2023 (Approval No. 2023(16173)), as the Dutch law on Research Involving Human Subjects Act (WMO) did not apply. Coaches provided permission, and all participants gave written informed consent. Coaches and participants were not financially compensated for their participation.

### Participants

Male volleyball players, aged 16 to 21 years, from the Dutch national talent youth team were eligible to participate. No exclusion criteria were applied.

### Study measures

#### Anthropometrics

Basic characteristics including age, dominant leg, height, and weight were collected via an athlete management system (AMS; Smartabase Athlete app version 1.33.0, Fusion Sport, Milton Brisbane, Australia).

#### Patellar tendon stiffness

**Fig. 2:**
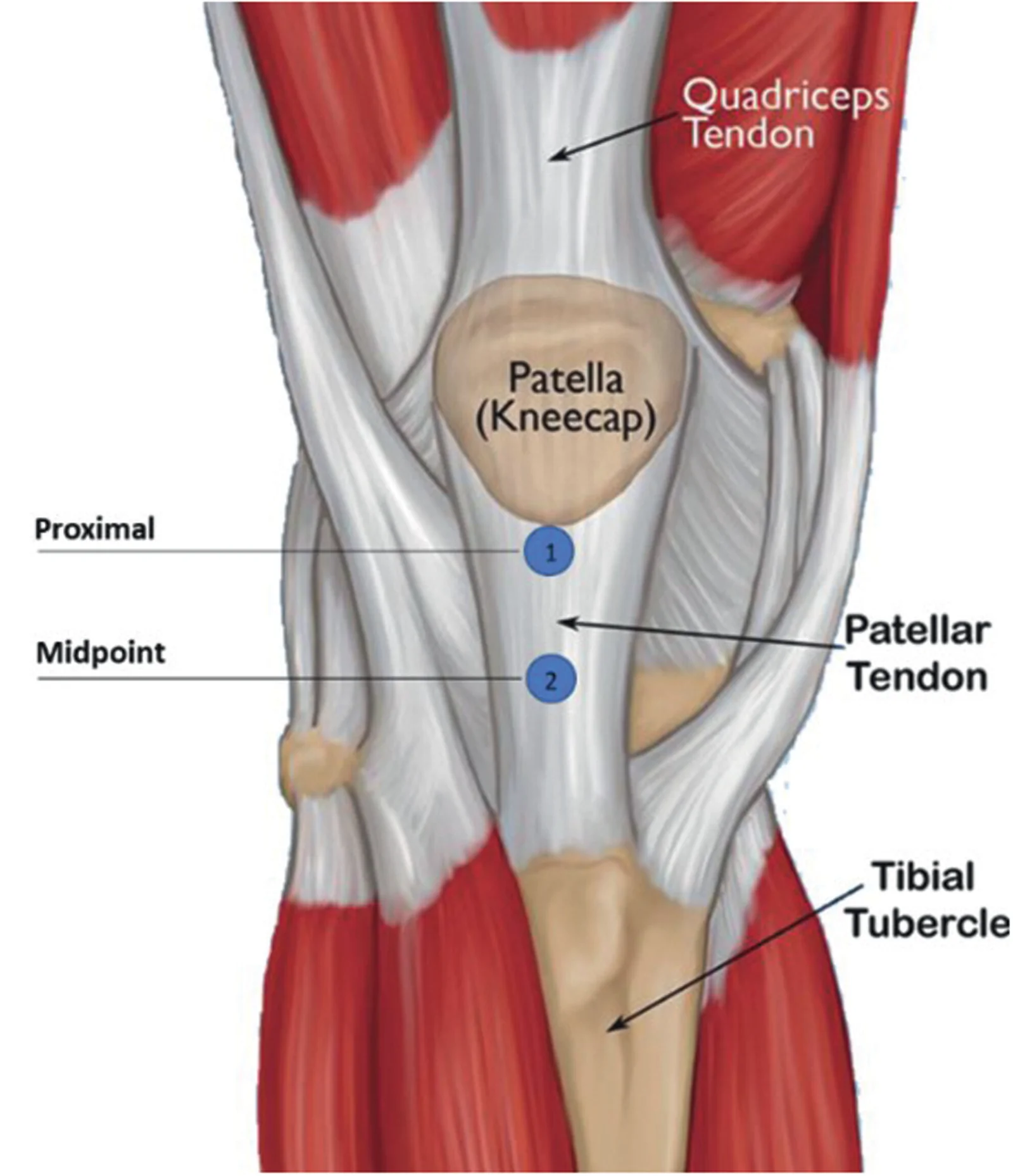
The two measurement locations on the tendon. Source: Reproduced and modified with permission from OrthoInfo. © American Academy of Orthopaedic Surgeons. https://www.orthoinfo.org/

PT stiffness (N/m) was measured weekly, before and after a training session, with the handheld MyotonPRO (Myoton, Tallinn, Estonia). Measurements were standardized (supine, feet fixed, and knee at 90◦) at two tendon sites: directly below the apex patella (proximal) and at the midpoint between the patella and tibial tuberosity (midpoint; Fig. 2 ). 14​ According to the measurement location and timepoint, measurements were performed once, with the device providing the average of five taps. Only one specifically trained researcher, not related to the team, performed the measurements.

#### Load monitoring

The internal load was assessed using session-rating of perceived exertion (RPE) rated on a modified Borg CR-10 scale (0–10; RPE x duration) recorded in the AMS SmartaBase app. 17​ The weekly training load was obtained by summing all session-RPE scores for the week. The external load was measured as the jump frequency with an activity tracker (KINEXON inertial measurement unit [IMU], Precision Technologies, Munich, Germany), validated for volleyball players. 18​

#### Pain

Weekly single-leg decline squats (SLDS) on a 25° board to 60° knee flexion were performed. Pain was rated on a 0–10 visual analogue scale (VAS). 19​

#### Function

Participants completed the Dutch Victorian institute of sport assessment–patella (VISA-P) questionnaire, assessing pain, function and sport activity, weekly in the AMS SmartaBase app. 20​ Participants had to fill out the questionnaire before the Tuesday training session. A score of 100 indicates no pain, maximal function and maximum ability to perform sport-related activities.

#### Adverse events

Adverse events were defined as events requiring the participant to seek additional healthcare within or outside sports setting or to use analgesic medication. Serious adverse events were defined as events resulting in hospitalization, surgery, permanent or temporary physical disability, life-threatening complications, or any other event considered to pose significant health risks. 21​ Events were recorded weekly in the AMS SmartaBase app.

#### Feasibility analyses

Measures included willingness to participate, retention, measurement completeness, and adverse events. Participation and retention rates were calculated as percentages of participating team members and withdrawals, respectively. Measurement completeness was assessed for stiffness (pre- and post-training), load, SLDS pain and VISA-P questionnaires as the proportion of completed to scheduled measurements. Each participant’s result was calculated individually, per week, and in total. Adverse events during the 12-week period were listed accordingly.

A priori success criteria were defined as greater than 80% for participation, retention, and measurement completeness; adverse events were comparable to previous studies. 22​ ​23​ Completeness was classified as excellent (≥ 80%), good-to-fair (70–80%), or poor (< 70%). Given the repetitive nature of weekly training sessions, the success criterion for load measurement completeness was set 10% lower. Results are presented in percentages and portions, with ranges.

Presentation of pilot data. Graphs depicting the pre- and post-training stiffness of the dominant knee measured at midpoint of the PT, internal load, SLDS pain of the dominant knee, and VISA-P scores.

## Results

### Feasibility

#### Willingness to participate and retention

All 15 eligible players from the national male talent volleyball team enrolled, and none withdrew during the 12-week monitoring period ( Table 1 ).

**Table:** **Table 1** Summary of basic physical characteristics

Male athletes ( *n* =15)	Mean±SD
Age (y)	18.0±1.1
Height (cm)	197±10
Weight (kg)	87.1±9.6
Body mass index (kg/m ^2^ )	22.5±1.3
Dominant leg, left/right	13L/2R
Baseline VISA-P score	75±23

Abbreviations: SD, standard deviation; VISA-P, Victorian Institute of Sport Assessment-Patella.

#### Measurement completeness

Table 2 provides an overview of measurement completeness for all participants over the 12-week monitoring period, indicating the completeness against the predefined success criteria. None of the participants achieved total measurement completeness above the success criteria (see **Supplementary Table S1, available in the online version only** for an overview of the measurement completeness per week). Of the 12-week period, 5 weeks had a measurement completeness above 80%. In week 9, no post-training measurements took place due to other training obligations immediately after the training session, and in week 11, participants were absent since no training took place on the measurement location, hindering further data collection.

**Table:** **Table 2** Measurement completeness for stiffness, load, pain, and function separated per participant over the 12-week monitoring period

Abbreviations: SLDS, single-leg decline squat; VISA-P, Victorian institute of sport assessment-patella.

Colors define excellent feasibility (white), good-to-fair feasibility (light grey) and poor feasibility (dark grey). Results are shown in percentage and the amount of performed measurements/total amount of planned measurements.

##### Stiffness

During the 12-week monitoring period, 72% (129 out of 180, range 17–92%) of stiffness measurements were completed before training, and 66% (118 of 180, range 17–83%) post-training. Six out of 15 participants met the 80% success criterion for pre-training measurements; however, only two participants achieved the success criterion for post-training measurements.

##### Internal and external loads

For internal load (RPE×duration), the average completeness was 52% (range: 12–87%), with 4 of 15 participants meeting the 70% success criterion ( Table 2 ). For the external load (jump frequency), the completeness was 38% (range 22–48%), and none of the participants met the predefined success criterion.

In terms of weekly completeness ( **Supplementary Tables S2 and S3** , available in the online version only), the internal load completeness exceeded the 70% success criterion in 3 of 12 weeks and external load in 2 weeks.

##### Pain

On average, the overall completeness of pain measurements was 71% (range: 17–92%). Six of 15 participants achieved the pain measurement completeness above 80%. The pain measurements were linked to the stiffness measurements before training, since both took place at the same time and same location.

##### Function

The overall questionnaire response rate was 68% (range: 17–92%). In 5 of the 15 participants, the questionnaire response rate was above the 80% success criterion.

#### Adverse events

Throughout the monitoring period, no serious adverse events and no usage of analgesia were reported by participants. Two cases of viral illness were reported causing both participants to miss 1 week of measurements.

### Pilot outcomes

**Fig. 3:**
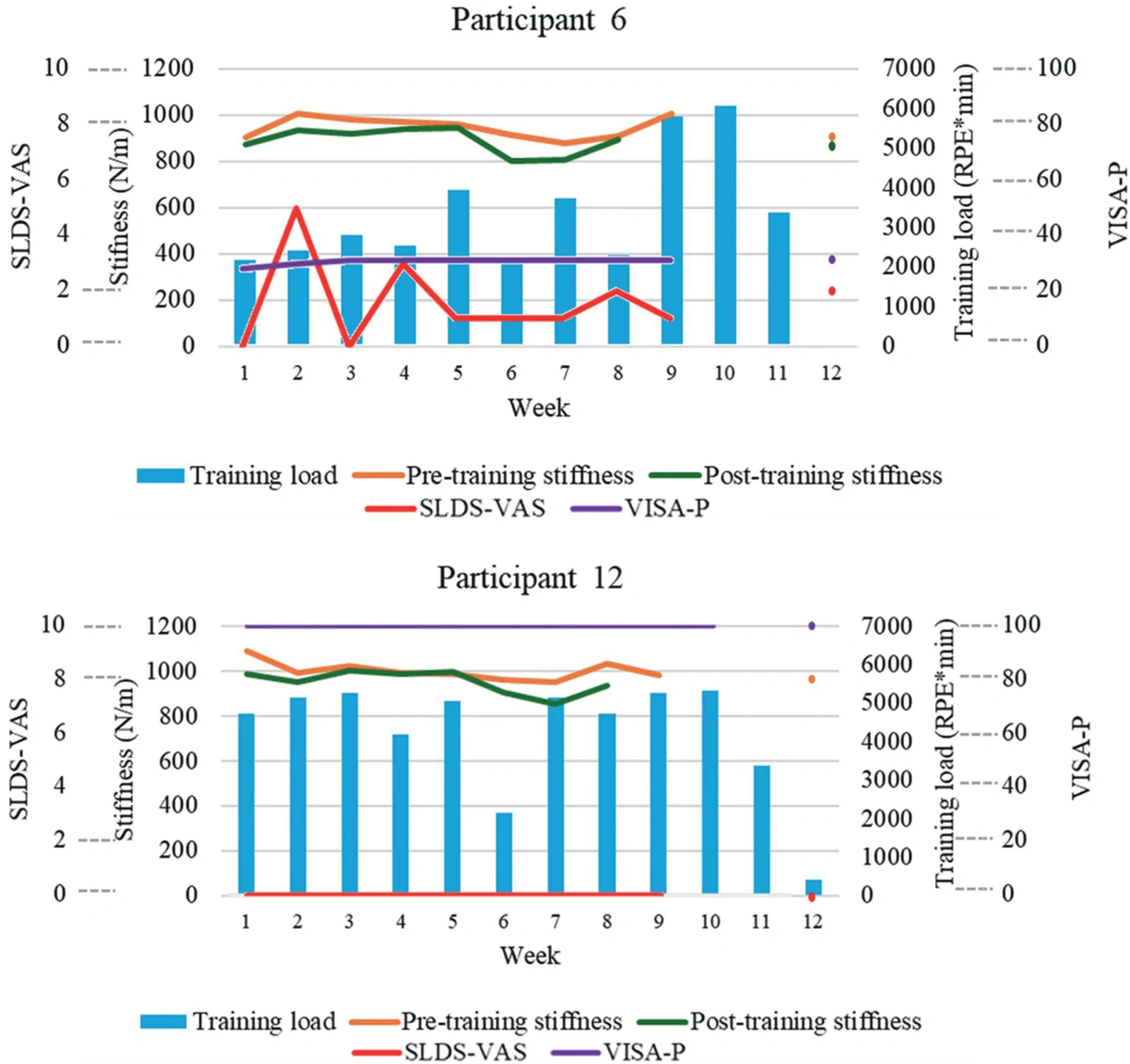
The individual results of participant 6 ( **a** ) and 12 ( **b** ) are shown as an example of how the output of a monitoring study would look like. Graph includes the pre- and post-training PT stiffness, the internal training load, the pain score after the SLDS and VISA-P score. RPE, rating of perceived exertion; SLDS-VAS, single-leg decline squat, visual analogue scale score.

In Fig. 3 , we present the graphs with individual data of the two participants with the highest measurement completeness (although below the success criterion), in order to visualize the insight you can get as the athlete/coach/healthcare professional when these parameters are accurately monitored over a longer period of time. Data collection was incomplete because several measurement sessions were missed, resulting in varying levels of data availability across participants and outcome measures. Consequently, a full longitudinal data set was not obtained for every participant, and some outcomes could not be reported consistently across all individuals.

## Discussion

Collecting a complete data set of PT stiffness, load, pain and function appeared to be challenging in this prospective observational monitoring study of talented volleyball players. Especially, measurement completeness for the load was far below the specified success criteria. Several modifications are necessary in future monitoring (studies) to obtain more complete data, aiming for better insight into the stiffness in relation to other factors and determining its role in the prevention of patellar tendinopathy.

### Feasibility outcomes

#### Willingness to participate and retention

The willingness of all team members to participate and the 100% retention rate reflect high participant acceptance of the protocol, suggesting that the monitoring approach was well tolerated. Using existing connections with coaches and medical staff from the national volleyball team contributed to these results, and monitoring a whole team on a fixed day of the week. Measuring on a fixed day of the week within a team setting may have contributed to high retention through clarity and positive group dynamics.

#### Measurement completeness

In order to achieve an 80% measurement completeness, procedural adjustments are needed in future studies. Although exact reasons for missing data were not systematically assessed, measurement completeness for stiffness, load, pain, and function was influenced by multiple factors like presence at training, motivation, communication, work- or social-related stress, study burden, and the presence of concurrent health-related conditions. Completeness of measurements performed on participants—such as tendon stiffness, SLDS pain score, and jump height and frequency—was hindered primarily by logistical, presence-related, procedural, and technical/instructional constraints rather than by device failure.

Measuring within a team setting did offer some logistical advantages because athletes are typically present at the same time and place during training, which facilitates data collection. In addition, we included talent athletes, who are known to have a higher commitment to attend to all training sessions compared to recreational athletes. 24​ However, due to logistic reasons, measurements could only be performed at the national Olympic center. Participants did not only train in the Olympic center but also with their local clubs at a different location with no measurement devices present. Consequently, it was not possible to collect complete weekly data. For example, in week 9, it was impossible to perform the post-training measurement because the participants immediately had to proceed to another scheduled training session at a different location. In week 11, all participants were absent due to a change in schedule that was not communicated to the researcher, hindering data collection for that week. Other reasons included participants who arrived too late for training sessions or had to leave immediately after training because of academic or social commitments resulting in missing data. This highlights the importance of good communication and emphasizing the importance of the measurements. When coaches or other staff members could perform the measurements, the performance of the measurements could become a part of the training routine and eventually, a habit.

The availability of only one tendotonometry device limited the speed at which measurements could be performed, resulting in skipped measurements when time constraints arose. Although tendotonometry is a quick method, measurements took on average 5 minutes per participant (including participant positioning, tendotonometry, and the SLDS), having the other participants waiting. These issues collectively made post-training stiffness assessments particularly challenging, which can be solved by using multiple measurement devices in future projects.

The IMUs were unintentionally left off (either not worn by the participants or not activated by the coaches), had depleted batteries, or failed to establish a connection, although the latter occurred only rarely. Notably, external load data were incompletely recorded during the initial weeks of the study, underscoring the importance of thorough education and instruction of both athletes and coaches on the consistent device use at the study onset.

Altogether, these findings suggest that future studies could benefit from an embedded scientist, coach or other staff member to perform the measurements, consistent use of IMUs, clear study instructions, and the use of multiple measurement devices.

Due to issues related to motivation, compliance and communication, measurements completed by participants like questionnaires and RPE scores were particularly prone to missing data, and resulted in a decrease in measurement completeness over time. Although previous studies have frequently monitored load using RPE, which is considered easy to use, feasibility in the present study could not be confirmed. 25​ ​26​

A significant challenge in ensuring compliance with participant-reported data is the perceived disparity between providing data and its practical applications and feedback. 26​ ​27​ When athletes do not observe meaningful training adjustments, resulting from their reported data, compliance tends to decline rapidly, often falling below 60% within 4 to 8 weeks. 26​ Previous studies have reported even lower response rates, with compliance dropping below 49% or as low as 14% after 4 weeks of monitoring. 27​ These findings highlight that athletes become more engaged in data collection when they see that the monitored data are actively used to adjust their training load, reinforcing the relevance of their efforts and increasing their motivation to contribute.

Completing questionnaires after every training session is a considerable burden which also does not contribute to data completeness. This emphasizes the need for sustained motivation strategies, repeated reminders and digital prompts, especially when monitoring is performed during longer periods like a whole season. Athletes often become more engaged with monitoring when they have experienced a previous injury. 28​ Structured educational sessions on (tendon) health, injury prevention, and the relevance of monitoring and appropriate timely feedback based on the collected data may enhance long-term compliance.

Finally, effective communication between researchers, coaches, and athletes is essential for optimizing measurement performance and study adherence (e.g., sharing practical information like changes in the training schedule). 29​ Strong researcher–coach–athlete relationships improve this communication. In addition, coach buy-in is likely a key determinant of the successful implementation of monitoring programs. Coaches often have a substantial influence over athletes’ daily routines and can strongly encourage, or even require, the completion of monitoring activities. In addition, coaches can educate athletes about the purpose and potential impact of monitoring tendon health. When coaches perceive value in the collected data and actively support the monitoring process, athlete engagement and compliance may improve considerably. Previous research has demonstrated that a higher quality communication between athletes, coaches, and medical staff is associated with reduced injury rates, emphasizing the importance of within multidisciplinary team communication in both research and applied sports (medicine) settings. 30​

Altogether, future monitoring (studies) should focus on clear communication between researchers, coaches, and athletes. By providing timely personalized feedback, athletes can be kept motivated to complete questionnaires and report subjective scores.

### Pilot outcomes

Individual data illustrate potential insights from near-complete data sets, showing a variation in stiffness and SLDS pain over time. This could help to identify a potential association between pain and stiffness over a longer period of time. Although previous studies have reported good intra- and inter-rater reliability of tendotonometry, the construct validity of tendon stiffness measurements remains unclear. 14​ As a result, observed fluctuations in stiffness should be interpreted with caution, as it is uncertain to what extent they reflect meaningful biological changes within the tendon. In this pilot study, reporting of the training load output was variable and since the actual (internal and external) load was incomplete, it is impossible to draw any preliminary conclusions about the stiffness and load relationship.

The interpretation of the present findings may benefit from consideration of previous feasibility work investigating prospective monitoring strategies and decisions on which measurements to perform in athletes with a high risk of developing patellar tendinopathy. For example, the feasibility of various clinical and biomechanical outcome measures has been examined in a cohort of jumping athletes, highlighting important considerations for longitudinal study designs in this population. 31​ Their work demonstrated that, while multiple measures are available to assess patellar tendinopathy, not all are equally feasible or suitable for repeated assessments over time in athletic cohorts. One example is the VISA-P questionnaire, which in the current study appeared to be less sensitive to short-term changes over time. Recent research has shown that the VISA-P has its limitations and that it is not robust enough for measuring symptom changes in patellar tendinopathy. 32​ Pain scores measured using the SLDS are more sensitive for assessing changes in patellar tendinopathy symptoms. 32​ The SLDS is also a tendon-specific and very quick and easy measure, making it particularly feasible for frequent monitoring in applied settings. Other methods for monitoring patellar tendinopathy include imaging techniques such as ultrasound, which provide structural information but are often limited by costs, availability, and operator dependency. Alternative functional measures such as the jump height or maximal quadriceps strength are more feasible in daily practice but are less specific to tendon properties. Compared to these approaches, tendotonometry offers a feasible option for the repeated assessment of tendon stiffness, although its added value beyond symptom monitoring (e.g., SLDS) and performance metrics still requires further investigations. 33​

Due to the large number of variables relating to stiffness, load, pain and function, it will be challenging not only to collect complete data but also to identify relevant changes and relationships across all measured outcomes. This might raise the question which measurements to perform on a weekly basis (like PT stiffness and SLDS) and which on a lower frequency (like VISA-P). The VISA-P questionnaire can for example be filled out only when changes in pain and/or function are monitored, reducing the burden of participation and increasing study compliance. This underscores the importance of carefully selecting outcome measures that are both sensitive to change ánd practical for regular monitoring in real-world sports settings. In this context, the current study aligns with the growing emphasis on feasible, athlete-friendly, holistic measurement approaches, but also highlights the need for the further refinement of longitudinal assessment strategies in PT research.

### Strengths and limitations

Our study is the first to investigate the feasibility of monitoring long-term changes in PT stiffness, load, pain and function in volleyball players. It also describes considerations and recommendations for improving data collection and completeness in future monitoring (studies) within a (elite) sports setting. We performed this study in a team of male talented volleyball players, a population with a high prevalence of patellar tendinopathy, which makes it a relevant group for monitoring PT health and preventing PT injuries.

A limitation of this study is that no semi-structured interviews were conducted limiting our ability to gain in-depth insight into participants’ and coaches’ perspectives on the missing data. However, valuable recommendations for future monitoring were provided based on the information collected from coaches and researchers. A second limitation is that it is difficult to precisely measure total load exposure, especially since the load is a broader concept than just the training load. Differences in load outside of training sessions might have been present between participants and within participants. Future studies could investigate tendon loading using innovative IMUs and/or elaborate questionnaires to obtain more insight into tendon loading in daily life. A third limitation of the current study is that we only included male participants. Although patellar tendinopathy is more prevalent in men, 34​ it is of interest to consider what happens in women when it comes to feasibility and whether there are potential sex differences in stiffness, load, pain, and function. This is of particular interest because research on tendon pathology has primarily focused on men. 35​ A fourth limitation relates to the context in which the monitoring protocol was implemented. The participating athletes were monitored within a highly structured environment, using an established AMS and maintaining close relationships with the research and support staff. Such conditions are not representative of other teams, sports, countries, or performance settings, where access to monitoring infrastructure, staff support, and athlete engagement may differ substantially. Consequently, the feasibility outcomes observed in this study, including compliance rates and data completeness, are not directly transferable to other sporting environments. Future research should therefore evaluate the feasibility of monitoring approaches across a broader range of sports and organizational contexts to determine their generalizability. A final limitation of the current study is that we did not monitor parameters such as fatigue, recovery and performance variability. Future studies could adopt an even more holistic approach to monitoring tendon characteristics over the course of a whole season, in order to establish associations between changes in training load and tendon health, and signs of tendon pathology. 36​

### Conclusions

Long-term monitoring of stiffness, load, pain and function in volleyball players seems to be important for the early detection and (load) management of patellar tendinopathy, as well as its prevention. This feasibility study shows that there are several challenges in obtaining a complete data set when monitoring in talented volleyball players. Communication between researcher–coach–athlete, feedback on collected data, education on importance of maintaining (tendon) health can be considered important factors to overcome these challenges.

Biographical note. Lotte van Dam works as a research coordinator at the university medical centre in Utrecht, and works on her PhD in human movement sciences at the Rijksuniversiteit Groningen. She graduated with a master’s in Human Nutrition and Health at the Wageningen University and a master’s in Human Movement Sciences at the Vrije Universiteit Amsterdam (VU). During these master’s and while working, she has done many different studies with (top) athletes, with a focus on nutrition, sports, and health.

Rieneke Terink is the principal investigator. She studied cell biology at Wageningen University and used to be a professional competitive swimmer (Ek’s, Wk’s, and WorldCups). She received her PhD degree from Wageningen University on the topic: “effect of exercise on nutritional status, and the stress and immune response”. She currently works at Hospital Gelderse Vallei, initiating and leading (new) studies focused on nutrition, health, and exercise. At Sports Valley, her role revolves around further developing nutritional concepts in sports medical care, disseminating knowledge and fostering new collaborations. With significant experience in conducting intervention studies with athletes, she specialized in data collection on physical performance, including energy metabolism, movement and tendon measurements.

Prof. dr. I (Inge) van den Akker-Scheek is a human movement scientist and associate professor at the department of Orthopedics of the University Medical Center Groningen, University of Groningen, the Netherlands. Her research focuses on optimising treatment and rehabilitation, development and evaluation of (patient reported) outcome measures, and the development and implementation of interventions to stimulate physical activity before, during and after treatment of patients with musculoskeletal disorders, in particular patients with osteoarthritis or tendinopathy.

Prof. dr. J. (Hans) Zwerver is Professor of Sports and Exercise Medicine at the Department of Human Movement Sciences of the University Medical Center Groningen and also works as a sports physician at the department of Sports Medicine (Sports Valley) in hospital Gelderse Vallei. His research focuses on the prevention, diagnosis, and treatment of sports injuries (such as tendinopathy) and “healthy active aging.” He has performed several research project and trials in patellar tendinopathy and volleyball.

## Supplementary Material

Supplementary Material Zusatzmaterial
