# Supplementary material for: Monitoring Patellar Tendon Stiffness in Relation to Load, Function and Pain
Source: Sports Med Int Open. 2026 Aug 31;10:a29307880. doi: 10.1055/a-2930-7880 (PMC13551455; doi:10.1055/a-2930-7880)
Supplement: Supplementary file 1 [file 10-1055-a-2930-7880_29336459.pdf]

Table S1 Total data collected per week, shown in percentages

Week	Stiffness before	Stiffness after	SDLS	VISA-P	Total
1	87% (13/15)	87% (13/15)	87% (13/15)	87% (13/15)	87% (52/60)
2	93% (14/15)	93% (14/15)	87% (13/15)	80% (12/15)	88% (53/60)
3	80% (12/15)	80% (12/15)	80% (12/15)	67% (10/15)	77% (46/60)
4	87% (13/15)	87% (13/15)	87% (13/15)	73% (11/15)	83% (50/60)
5	87% (13/15)	87% (13/15)	87% (13/15)	87% (13/15)	87% (52/60)
6	87% (13/15)	87% (13/15)	87% (13/15)	87% (13/15)	87% (52/60)
7	60% (9/15)	60% (9/15)	60% (9/15)	60% (9/15)	60% (36/60)
8	93% (14/15)	93% (14/15)	93% (14/15)	80% (12/15)	90% (54/60)
9	73% (11/15)	0% (0/15)	73% (11/15)	47% (7/15)	48% (29/60)
10	40% (6/15)	33% (5/15)	40% (6/15)	60% (9/15)	43% (26/60)
11	0% (0/15)	0% (0/15)	0% (0/15)	13% (2/15)	3% (2/60)
12	73% (11/15)	73% (11/15)	73% (11/15)	73% (11/15)	73% (44/60)

The total amount of times parameters were collected per week. Maximum was 15 for the 15 participants. Abbreviations: SLDS, single-leg decline squat, VISA-P, Victorian institute of sport assessment – patella

Table S2. Internal load feasibility separated per week for each participant

Participant	Week 1	2	3	4	5	6	7	8	9	10	11	12	Total weeks >4
1	4/5	2/5	1/5	2/5	3/5	1/5	2/5	0/5	0/5	0/5	0/5	0/5	1/12
2	5/5	5/5	5/5	5/5	2/5	0/5	2/5	3/5	0/5	3/5	0/5	0/5	4/12
3	0/5	0/5	2/5	1/5	5/5	2/5	4/5	5/5	5/5	4/5	3/5	0/5	5/12
4	4/5	4/5	4/5	3/5	3/5	3/5	5/5	1/5	2/5	5/5	1/5	0/5	5/12
5	5/5	4/5	3/5	2/5	4/5	2/5	3/5	1/5	2/5	3/5	0/5	0/5	3/12
6	3/5	4/5	4/5	4/5	5/5	3/5	4/5	3/5	5/5	5/5	3/5	0/5	7/12
7	3/5	3/5	2/5	0/5	0/5	0/5	0/5	0/5	0/5	0/5	0/5	0/5	0/12
8	3/5	0/5	1/5	0/5	0/5	0/5	0/5	0/5	1/5	1/5	1/5	0/5	0/12
9	4/5	3/5	5/5	4/5	2/5	1/5	1/5	0/5	0/5	1/5	0/5	0/5	3/12
10	4/5	5/5	4/5	3/5	5/5	2/5	5/5	0/5	0/5	0/5	0/5	0/5	5/12
11	5/5	4/5	3/5	4/5	5/5	2/5	5/5	5/5	5/5	2/5	0/5	1/5	7/12
12	5/5	5/5	5/5	5/5	5/5	3/5	5/5	5/5	5/5	5/5	3/5	1/5	9/12
13	5/5	5/5	5/5	5/5	5/5	3/5	4/5	0/5	1/5	5/5	3/5	1/5	7/12
14	5/5	5/5	3/5	4/5	5/5	3/5	2/5	5/5	0/5	2/5	0/5	0/5	5/12
15	3/5	5/5	5/5	5/5	5/5	3/5	5/5	5/5	5/5	5/5	3/5	1/5	8/12
Total	58/75	54/75	52/75	47/75	54/75	28/75	47/75	33/75	31/75	41/75	17/75	4/75	
%	77	72	69	63	72	37	63	44	41	55	23	5	

Internal load feasibility measured using the RPE*training duration. Colours define excellent feasibility (white), good-to-fair feasibility (light grey) and poor feasibility (dark grey). Results are shown in amount of performed measurements / total amount of planned measurements. The total amount of excellent weeks is shown in the right column. The total percentage and fraction of measurements performed / total amount of planned measurements per week is shown in the bottom two rows.

Table S3. External load feasibility separated per week for each participant

Participant	Week 1	2	3	4	5	6	7	8	9	10	11	12	Total weeks >4
1	1/5	1/5	0/5	1/5	1/5	3/5	4/5	2/5	5/5	3/5	0/5	2/5	2/12
2	1/5	1/5	1/5	1/5	1/5	2/5	4/5	3/5	5/5	5/5	0/5	1/5	3/12
3	1/5	1/5	0/5	1/5	1/5	3/5	3/5	1/5	5/5	4/5	2/5	0/5	2/12
4	1/5	1/5	1/5	1/5	1/5	3/5	5/5	3/5	5/5	3/5	1/5	0/5	2/12
5	1/5	1/5	1/5	1/5	1/5	3/5	4/5	2/5	5/5	5/5	2/5	2/5	3/12
6	1/5	1/5	1/5	1/5	1/5	3/5	5/5	2/5	5/5	3/5	2/5	0/5	2/12
7	0/5	0/5	1/5	1/5	1/5	2/5	4/5	2/5	5/5	0/5	0/5	0/5	2/12
8	1/5	0/5	0/5	1/5	1/5	2/5	1/5	1/5	4/5	2/5	1/5	0/5	1/12
9	0/5	1/5	1/5	1/5	1/5	3/5	5/5	4/5	5/5	5/5	2/5	1/5	4/12
10	1/5	0/5	1/5	1/5	0/5	3/5	2/5	1/5	4/5	0/5	0/5	0/5	1/12
11	1/5	0/5	1/5	1/5	1/5	2/5	3/5	2/5	5/5	4/5	2/5	2/5	2/12
12	1/5	1/5	1/5	1/5	1/5	3/5	5/5	3/5	5/5	3/5	2/5	0/5	2/12
13	1/5	1/5	1/5	1/5	1/5	3/5	4/5	4/5	5/5	4/5	1/5	0/5	4/12
14	1/5	0/5	1/5	0/5	1/5	2/5	3/5	3/5	5/5	4/5	2/5	2/5	2/12
15	0/5	0/5	1/5	1/5	1/5	3/5	5/5	4/5	5/5	3/5	1/5	0/5	3/12
Total	12/75	9/75	12/75	14/75	14/75	40/75	57/75	37/75	73/75	48/75	18/75	10/75	
%	16	12	16	19	19	53	76	49	97	64	24	13	

External load feasibility measured using the jump frequency of a training. Colours define excellent feasibility (white), good-to-fair feasibility (light grey) and poor feasibility (dark grey). Results are shown in amount of performed measurements / total amount of planned measurements. The total amount of excellent weeks is shown in the right column. The total percentage and fraction of measurements performed / total amount of planned measurements per week is shown in the bottom two rows.
